# Evaluation of the CHG index for identifying metabolic dysfunction associated steatotic liver disease: evidence from two independent Asian non-obese populations

**DOI:** 10.3389/fnut.2026.1757164

**Published:** 2026-02-20

**Authors:** Jun Chen, Qiuyi Liang, Zeru Chen, Wang Liu, Xiaomi Chen, Huan Li, Dongjie Huang, Shuyue Zhou, Riken Chen, Junqi Ren, Xiaoling Wu, Jinhua Liang

**Affiliations:** The Second Affiliated Hospital of Guangdong Medical University, Zhanjiang, Guangdong, China

**Keywords:** CHG index, glucose-lipidmetabolism, identification, insulin resistance, metabolic dysfunction-associated steatotic liver disease, ultrasonography

## Abstract

**Background:**

Metabolic dysfunction-associated steatotic liver disease (MASLD) is highly prevalent and is driven by coupled abnormalities in glucose and lipid metabolism. Single metabolic markers such as total cholesterol (TC), fasting blood glucose (FBG), or high-density lipoprotein cholesterol (HDL-C) reflect isolated pathways and often show limited predictive value. The cholesterol, high-density lipoprotein, and glucose (CHG) index integrates lipid and glycaemic information and may better capture MASLD-related metabolic risk.

**Methods:**

Two de-identified health-check cohorts from the Dryad repository were analyzed: a Chinese derivation cohort from Wenzhou Medical Center (2010–2014; *n* = 16,173) and a Japanese validation cohort from the NAGALA study (2004–2015; *n* = 11,981). CHG was calculated from fasting TC, FBG, and HDL-C. MASLD was diagnosed by abdominal ultrasonography according to national society criteria. Associations were examined with multivariable logistic regression (CHG continuous and quartiles) and restricted cubic splines. Discrimination was compared with TC, FBG, HDL-C, and the triglyceride–glucose (TyG) index using receiver operating characteristic (ROC) curves and area under the curve (AUC). Incremental value beyond baseline models was assessed by net reclassification improvement (NRI) and integrated discrimination improvement (IDI).

**Results:**

Higher CHG was consistently associated with MASLD in both populations, showing clear dose–response gradients. Participants in the highest CHG quartile had approximately fourfold higher adjusted odds of MASLD than those in the lowest quartile. CHG yielded the highest AUC among all markers in both cohorts and improved baseline model discrimination and reclassification, with positive NRI and IDI.

**Conclusion:**

The CHG is robustly and dose-dependently associated with MASLD and demonstrates superior, reproducible discriminative performance compared with traditional single markers across two independent Asian cohorts. CHG may serve as a practical, low-cost tool for MASLD screening and risk stratification at baseline health check-ups.

## 1 Introduction

Metabolic dysfunction-associated steatotic liver disease (MASLD) has become the leading chronic liver disorder worldwide (1), currently affecting roughly 38% of adults, and its burden continues to increase each year (2–6). Beyond hepatic fat accumulation, MASLD represents a systemic metabolic condition. Patients, especially those who progress to metabolic dysfunction-associated steatohepatitis (MASH) or develop fibrosis, carry substantially higher risks of type 2 diabetes, cardiovascular events, chronic kidney disease, and several extrahepatic malignancies (3, 7–9). Given its high prevalence and serious complications, early identification of individuals more likely to have MASLD at routine health check-ups is essential, creating a pressing need for accessible screening and risk stratification tools.

Although diagnostic frameworks define MASLD by the presence of hepatic steatosis together with cardiometabolic risk factors (10), the disease is fundamentally driven by intertwined disturbances in glucose and lipid homeostasis. Conventional single laboratory measures, including total cholesterol (TC), fasting blood glucose (FBG), and high-density lipoprotein cholesterol (HDL-C), reflect only one aspect of metabolism at a time. Consequently, these isolated markers often fail to represent the integrated metabolic environment that promotes MASLD and may show suboptimal performance for identifying MASLD.

The cholesterol, high-density lipoprotein, and glucose (CHG) index integrates lipid and glycaemic information into a single composite biomarker. Prior studies indicate that HDL-C is negatively associated with MASLD, whereas TC and FBG are positively associated with MASLD (11, 12), suggesting that a combined indicator may better represent metabolic dysfunction relevant to hepatic steatosis. CHG was initially used in diabetes risk assessment (13) and has recently been proposed as a marker of metabolic dysfunction and insulin resistance (IR) (14). However, the association between CHG and MASLD, and whether CHG provides incremental discriminative value beyond traditional single markers, remains under-explored.

Therefore, this study aimed to evaluate CHG for identifying prevalent MASLD detected at baseline health check-ups in two independent cohorts (Chinese derivation and Japanese validation). We assessed the association between CHG and MASLD and compared its discriminative performance with TC, FBG, HDL-C, and the triglyceride–glucose (TyG) index. This design enables a MASLD-specific, head-to-head benchmarking framework with external validation in two Asian populations restricted to body mass index (BMI) ≤ 25 kg/m^2^.

## 2 Materials and methods

### 2.1 Data source

All analyses were based on de-identified data downloaded from the Dryad public repository^1^, an open-access archive that permits secondary use of deposited datasets.

#### 2.1.1 Chinese derivation cohort

The primary cohort was a retrospective sample derived from the health examination database of Wenzhou Medical Center, People’s Hospital of Wenzhou (15). Consecutive adults who attended routine health checkups between January 2010 and December 2014 were eligible, without any pre-screening criteria. A total of 33,135 examinees completed a standardized assessment that included abdominal ultrasonography of the liver.

Participants were excluded if they had high alcohol intake, current use of antihypertensive, glucose-lowering or lipid-lowering medication, a documented etiology of chronic liver disease, or baseline low-density lipoprotein cholesterol (LDL-C) > 3.12 mmol/L or BMI > 25 kg/m^2^. Individuals with missing key covariates were excluded. The final analytic sample comprised 16,173 participants.

All data were permanently anonymized prior to analysis, and the study was conducted in accordance with the Declaration of Helsinki. Ethical approval was obtained from the Ethics Committee of the People’s Hospital of Wenzhou (15). According to Article 32 of the National Health Commission guidance on ethical review of human life science and medical research (Guo Wei Ke Jiao Fa [2023] No. 4), the use of retrospective, de-identified records qualified for a waiver of informed consent. Unique health checkup codes, rather than personal identifiers, were used throughout to protect participant privacy.

#### 2.1.2 Japanese validation cohort

The NAGALA cohort was used as an external dataset for validation. This cohort comprises adults who participated in health screening programs at Murakami Memorial Hospital between 2004 and 2015 (16). Individuals were excluded if any of the following applied: FBG at or above 6.1 mmol per liter, missing essential variables, a prior diagnosis of liver disease, alcohol consumption of 60 g per day or more in men or 40 grams per day or more in women, or current use of any medication. To harmonize the inclusion/exclusion criteria across the two cohorts, we additionally excluded participants with BMI > 25 kg/m^2^ in the Japanese cohort (*n* = 2,261), leaving 11,981 individuals for the final analysis. The study protocol was approved by the ethics committee of Murakami Memorial Hospital, and written informed consent was obtained from all participants.

### 2.2 Definitions of CHG and MASLD

The exposure variable was the CHG index, calculated as (17, 18):

$$\mathrm{CHG}=\ln\frac{\mathrm{TC}\;(\mathrm{mg}/\mathrm{dL})\times \mathrm{FBG}\;(\mathrm{mg}/\mathrm{dL})}{2\times \mathrm{HDL}-C\;(\mathrm{mg}/\mathrm{dL})}$$

This study was designed as a retrospective cross-sectional analysis of health check-up data. The study endpoint was prevalent MASLD detected at baseline health check-ups. In the Chinese cohort, hepatic steatosis was assessed according to the recommendations of the Chinese Society of Hepatology (19), and in the Japanese cohort according to the Japanese Society of Gastroenterology (20). Both guidelines rely on abdominal ultrasonography and apply the same operational rule: hepatic steatosis was diagnosed when at least two of the following three findings were present: diffuse hepatic hyperechogenicity, increased liver–kidney echo contrast, and posterior beam attenuation with poor visualization of deep intrahepatic vessels.

The MASLD was then defined in a guideline-consistent operational manner as ultrasound-detected hepatic steatosis accompanied by at least one cardiometabolic risk condition assessed at baseline, including overweight (BMI ≥ 23.0 kg/m^2^), dysglycaemia/diabetes (fasting blood glucose ≥5.6 mmol/L or self-reported diabetes when available), lipid abnormality (triglycerides ≥1.7 mmol/L or low HDL-C < 1.0 mmol/L in men or <1.3 mmol/L in women), or elevated blood pressure (systolic blood pressure ≥130 mmHg or diastolic blood pressure ≥85 mmHg). The same operational definition was applied consistently in both the Chinese and Japanese cohorts.

### 2.3 Data collection procedures and measurement protocols

#### 2.3.1 Chinese cohort

In the Chinese cohort, data were obtained from a standardized hospital-based health examination program (21). After an overnight fast of at least 8 h, venous blood samples were collected and analyzed in the hospital laboratory using uniform procedures. Baseline sociodemographic characteristics and medical histories were recorded for each participant. Height and weight were measured, and body mass index was calculated as an index of overall adiposity. Blood pressure was measured by trained staff with the participant seated and at rest in a quiet room. Metabolic status was assessed using a biochemical panel that included liver and kidney function tests, FBG and serum lipids. All biochemical measurements were performed on an Abbott AxSYM automated analyzer according to standardized laboratory protocols.

#### 2.3.2 Japanese cohort

In the Japanese cohort, information on sociodemographic characteristics, lifestyle behaviors and medical history was collected using a structured questionnaire covering smoking status, alcohol consumption, habitual physical activity, previous illnesses and current medication use (22). Waist circumference, height, weight and blood pressure were measured by trained nurses or technicians following a uniform anthropometric protocol. After at least 8 h of fasting, venous blood was drawn and processed on an automated biochemical analyzer to determine liver function tests, glucose-related indices and lipid profiles. Smoking and alcohol intake were categorized into three levels, whereas habitual exercise and diabetes status were coded as binary variables.

### 2.4 Statistical analysis

Participants were classified as MASLD cases if hepatic steatosis was present on ultrasonography and at least one cardiometabolic risk condition was met according to the above criteria; otherwise, they were classified as non-MASLD. Baseline characteristics were summarized using distribution-appropriate statistics: variables following an approximately normal pattern are expressed as means with standard deviations, whereas non-normal measures are described by medians and interquartile ranges. For qualitative characteristics, results are shown as numbers and percentages. Between-group differences were evaluated with methods matched to data type and shape, applying chi-square tests to categorical outcomes, two-sample *t*-tests to normally distributed continuous measures, and Mann–Whitney *U* tests to continuous measures with skewed distributions.

To estimate the association between CHG and MASLD, CHG was analyzed in multivariable logistic regression as both a continuous exposure and a quartile-based categorical exposure. Three hierarchical models were constructed: an unadjusted model including CHG only; a partially adjusted model controlling for age, sex, and BMI; and a fully adjusted model further accounting for major metabolic covariates and liver and kidney function indices. Effect estimates are shown as odds ratios with 95% confidence intervals. Trends across quartiles were tested by assigning each quartile its median CHG value and modeling this variable continuously.

Potential non-linear dose–response relationships were examined using restricted cubic spline (RCS) functions within the fully adjusted framework. Four knots were placed at the 5th, 35th, 65th, and 95th percentiles of the CHG distribution. Non-linearity was assessed using likelihood ratio tests comparing models with and without spline terms. Subgroup analyses stratified by age and sex (and additionally by BMI, SBP, and DBP) were performed using fully adjusted logistic regression models, and effect modification was evaluated by adding CHG × subgroup interaction terms and reporting *p*-values for interaction. Discriminative performance of CHG was compared with the TyG index and traditional markers (TC, FBG, and HDL-C) using receiver operating characteristic (ROC) analysis, with the area under the curve (AUC) used for quantification. For incremental discrimination analyses, the baseline model was defined as the fully adjusted multivariable logistic regression model including all covariates adjusted for in the main multivariable analysis, but excluding CHG, and the “baseline + CHG” model was defined as the same model with CHG added. Predicted probabilities from these models were used to generate ROC curves and calculate AUC. The incremental discriminative contribution of CHG beyond baseline risk models was evaluated using net reclassification improvement (NRI) and integrated discrimination improvement (IDI). Subgroup analyses stratified by age and sex [and additionally by BMI, systolic blood pressure (SBP), and diastolic blood pressure (DBP)] were performed using fully adjusted logistic regression models; effect modification was evaluated by adding CHG × subgroup interaction terms and reporting *p*-values for interaction. All statistical analyses were conducted using R software (version 4.2.0), with the rms package for restricted cubic spline modeling, pROC for ROC analyses, and PredictABEL for NRI and IDI calculations; The FreeStatistics software (version 2.4.0) was used as an auxiliary platform. Two-sided *p*-values <0.05 were considered statistically significant.

## 3 Results

### 3.1 Baseline population features and differences

Among Chinese participants, 16,173 individuals met the inclusion criteria, and 2,032 were identified with MASLD (Table 1). Relative to those without MASLD, affected participants tended to be older, were more frequently men, and displayed greater adiposity and higher blood pressure, including both systolic and diastolic measures (all *p* < 0.001). The MASLD group also had a less favorable biochemical profile, with elevated TB, ALT, AST, GGT, LDL-C, TG, and CHG, alongside reduced Direct Bilirubin (DBIL) and HDL-C (all *p* < 0.001). By contrast, Blood Urea Nitrogen (BUN) showed no meaningful between-group differences (*p* > 0.05).

**Table 1:** Baseline characteristics of Chinese participants.

Variable	Overall (*N* = 16,173)	Non-MASLD (*N* = 14,141)	MASLD (*N* = 2,032)	*p*-value
Age (year)	43.23 ± 14.96	43.01 ± 14.91	44.75 ± 15.25	**<0.001**
Gender				**<0.001**
Female	7,690 (47.55)	6,814 (48.19)	876 (43.11)	
Male	8,483 (52.45)	7,327 (51.81)	1,156 (56.89)	
BMI (kg/m^2^)	21.38 ± 2.05	21.09 ± 1.98	23.39 ± 1.23	**<0.001**
SBP (mmHg)	120.73 ± 16.71	119.39 ± 16.40	130.02 ± 15.94	**<0.001**
DBP (mmHg)	72.81 ± 10.35	71.93 ± 10.07	78.89 ± 10.21	**<0.001**
ALP (U/L)	72.35 ± 23.22	71.20 ± 23.10	78.56 ± 22.93	**<0.001**
GGT (U/L)	22.00 (16.00, 31.00)	20.00 (16.00, 28.00)	32.00 (24.00, 48.75)	**<0.001**
ALT (U/L)	16.00 (12.00, 23.00)	16.00 (12.00, 21.00)	22.00 (17.00, 31.00)	**<0.001**
AST (U/L)	23.04 ± 9.53	22.65 ± 9.53	25.16 ± 9.25	**<0.001**
ALB (g/L)	44.40 ± 2.71	44.38 ± 2.70	44.53 ± 2.78	**0.032**
TB (μmol/L)	12.12 ± 4.95	12.07 ± 4.95	12.39 ± 4.96	**0.016**
DBIL (μmol/L)	2.29 ± 1.23	2.34 ± 1.24	1.99 ± 1.13	**<0.001**
BUN (mmol/L)	4.57 ± 1.37	4.56 ± 1.38	4.60 ± 1.26	0.185
Cr (mmol/L)	78.48 ± 25.68	77.30 ± 26.19	86.67 ± 20.03	**<0.001**
UA (μmol/L)	279.81 ± 85.92	272.34 ± 83.34	331.80 ± 85.68	**<0.001**
FBG (mg/dL)	92.65 ± 14.10	91.72 ± 12.99	99.08 ± 18.99	**<0.001**
TC (mg/dL)	178.81 ± 28.74	177.67 ± 28.30	186.75 ± 30.47	**<0.001**
TG (mg/dL)	95.66 (70.86, 133.75)	90.35 (68.20, 123.12)	159.43 (111.60, 217.23)	**<0.001**
HDL-C (mg/dL)	56.58 ± 14.07	57.62 ± 14.01	49.40 ± 12.21	**<0.001**
LDL-C (mmol/L)	2.26 ± 0.46	2.24 ± 0.46	2.40 ± 0.45	**<0.001**
CHG	5.00 ± 0.30	4.96 ± 0.29	5.23 ± 0.29	**<0.001**

Values are n (%) or mean ± SD or median (quartile).

BMI, Body mass index; DBP, Diastolic blood pressure; ALP, Alkaline phosphatase; SBP, Systolic blood pressure; GGT, γ-glutamyl transpeptidase; AST, Aspartate aminotransferase; TG, Triglyceride; ALB, albumin; ALT, Alanine aminotransferase; GLB, globulin; LDL-C, Low-density lipid cholesterol; BUN, Serum urea nitrogen; HDL-C, High-density lipoprotein cholesterol; Cr, Serum creatinine; TC, Total cholesterol; FBG, Fasting blood glucose; UA, uric acid; DBIL, Direct bilirubin; TB, Total bilirubin; CHG, Cholesterol, high-density lipoprotein, and glucose; MASLD, Metabolic dysfunction-associated steatotic liver disease.

*P*-value less than 0.05 is expressed in bold.

In the Japanese dataset, 11,981 participants were evaluated, of whom 945 had MASLD (Table 2). The same clinical pattern was observed. Individuals with MASLD were generally older, included a higher share of males, and had higher BMI, waist circumference, and both systolic and diastolic blood pressure compared with non-MASLD participants (all *p* < 0.001). Laboratory indices likewise indicated metabolic impairment, as MASLD cases exhibited higher GGT, ALT, AST, FBG, HbA1c, total cholesterol, triglycerides, LDL-C, and CHG, while HDL-C was lower (all *p* < 0.001).

**Table 2:** Baseline characteristics of Japanese participants.

Variable	Overall (*N* = 11,981)	Non-MASLD (*N* = 11,036)	MASLD (*N* = 945)	*P*-value
Age (year)	43.46 ± 8.99	43.22 ± 9.00	46.22 ± 8.30	**<0.001**
Gender				**<0.001**
Female	6,227 (51.97)	6,052 (54.84)	175 (18.52)	
Male	5,754 (48.03)	4,984 (45.16)	770 (81.48)	
BMI (kg/m^2^)	21.07 ± 2.14	20.87 ± 2.07	23.52 ± 1.13	**<0.001**
WC (cm)	73.78 ± 7.29	73.05 ± 7.01	82.20 ± 4.76	**<0.001**
SBP (mmHg)	111.82 ± 13.89	111.02 ± 13.56	121.15 ± 14.36	**<0.001**
DBP (mmHg)	69.68 ± 9.80	69.09 ± 9.56	76.58 ± 9.94	**<0.001**
GGT (U/L)	14.00 (11.00, 19.00)	13.00 (11.00, 18.00)	22.00 (16.00, 31.00)	**<0.001**
ALT (U/L)	16.00 (12.00, 21.00)	15.00 (12.00, 20.00)	25.00 (19.00, 34.00)	**<0.001**
AST (U/L)	17.57 ± 8.23	17.31 ± 8.27	20.58 ± 6.99	**<0.001**
FBG (mg/dL)	91.98 ± 7.32	91.54 ± 7.21	97.05 ± 6.66	**<0.001**
HbA1c (mmol/mol)	5.16 ± 0.31	5.15 ± 0.31	5.28 ± 0.33	
TC (mg/dL)	196.20 ± 33.09	195.07 ± 32.85	209.40 ± 33.08	**<0.001**
TG (mg/dL)	59.00 (41.00, 87.00)	57.00 (39.00, 82.00)	112.00 (79.00, 162.00)	**<0.001**
HDL-C (mg/dL)	58.35 ± 15.46	59.45 ± 15.30	45.47 ± 10.85	**<0.001**
Exercise				**0.003**
No	9,847 (82.19)	9,037 (81.89)	810 (85.71)	
Yes	2,134 (17.81)	1999 (18.11)	135 (14.29)	
Alcohol consumption				**0.004**
None	9,953 (83.07)	9,184 (83.22)	769 (81.38)	
Light	1,464 (12.22)	1,353 (12.26)	111 (11.75)	
Moderate	564 (4.71)	499 (4.52)	65 (6.88)	
Smoking status				**<0.001**
Never	7,610 (63.52)	7,186 (65.11)	424 (44.87)	
Past	2054 (17.14)	1790 (16.22)	264 (27.94)	
Current	2,317 (19.34)	2060 (18.67)	257 (27.2)	
Diabetes				**<0.001**
No	11,813 (98.60)	10,938 (99.11)	875 (92.59)	
Yes	168 (1.40)	98 (0.89)	70 (7.41)	
CHG	5.06 ± 0.32	5.03 ± 0.30	5.42 ± 0.27	**<0.001**

Values are n (%) or mean ± SD or median (quartile).

BMI, Body mass index; WC, Waist circumference; DBP, Diastolic blood pressure; SBP, Systolic blood pressure; GGT, γ-glutamyl transpeptidase; AST, Aspartate aminotransferase; TG, Triglyceride; ALT, Alanine aminotransferase; LDL-C, Low-density lipid cholesterol; HDL-C, High-density lipoprotein cholesterol; TC, Total cholesterol; FBG, Fasting blood glucose; DBIL, Direct bilirubin; TB, Total bilirubin; Hb1Ac, Glycosylated Hemoglobin, Type A1C; CHG, Cholesterol, high-density lipoprotein, and glucose; MASLD, Metabolic dysfunction-associated steatotic liver disease.

*P*-value less than 0.05 is expressed in bold.

Overall, both cohorts consistently indicate that MASLD is associated with poorer anthropometric and metabolic characteristics, and that CHG levels are higher in participants with MASLD in each population.

### 3.2 Higher CHG was associated with higher odds of prevalent MASLD in Chinese and Japanese cohorts

Across the Chinese and Japanese samples, increasing CHG levels tracked with progressively more prevalent MASLD cases (Table 3). Treating CHG as a continuous exposure, each 1-unit rise in CHG corresponded to a markedly higher MASLD risk after multivariable adjustment, with ORs of 1.82 (95% CI 1.67 to 1.98) in China and 1.60 (95% CI 1.42 to 1.80) in Japan. Categorizing CHG into quartiles yielded the same pattern of stepwise escalation. In the fully controlled Chinese model, relative to Q1, the odds increased to 1.47 (1.03 to 2.10) in Q2, 2.12 (1.51 to 2.97) in Q3, and 4.56 (3.29 to 6.33) in Q4, showing a strong dose gradient (*P*-trend <0.001). The Japanese cohort showed parallel increases versus Q1, with ORs of 2.21 (1.18 to 4.14), 3.95 (2.18 to 7.16), and 5.51 (3.03 to 10.03) for Q2 through Q4, respectively, again supporting a robust monotonic trend (*P*-trend <0.001).

**Table 3:** Association between CHG quartiles and prevalent MASLD in the Chinese and Japanese cohorts.

Variable	Model 1		Model 2		Model 3	
	OR (95% CI)	*P-*value	OR (95% CI)	*P-*value	OR (95% CI)	*P*-value
Chinese cohort						
CHG continuous	2.50 (2.38 ~ 2.64)	<0.001	1.99 (1.88 ~ 2.11)	<0.001	1.82 (1.67 ~ 1.98)	<0.001
CHG quartiles						
Q1	1.0 [Ref]		1.0 [Ref]		1.0 [Ref]	
Q2	2.8 (2.23 ~ 3.52)	<0.001	1.99 (1.57 ~ 2.53)	<0.001	1.47 (1.03 ~ 2.10)	0.034
Q3	5.4 (4.36 ~ 6.70)	<0.001	2.93 (2.34 ~ 3.67)	<0.001	2.12 (1.51 ~ 2.97)	<0.001
Q4	14.67 (11.95 ~ 18.02)	<0.001	6.73 (5.43 ~ 8.35)	<0.001	4.56 (3.29 ~ 6.33)	<0.001
*P-*trend	2.40 (2.27 ~ 2.53)	<0.001	1.89 (1.78 ~ 2)	<0.001	1.74 (1.59 ~ 1.89)	<0.001
Japanese cohort						
CHG continuous	3.73 (3.44 ~ 4.04)	<0.001	2.44 (2.22 ~ 2.69)	<0.001	1.60 (1.42 ~ 1.80)	<0.001
CHG quartiles						
Q1	1.0 [Ref]		1.0 [Ref]		1.0 [Ref]	
Q2	4.93 (2.71 ~ 8.97)	<0.001	2.52 (1.37 ~ 4.64)	0.003	2.21 (1.18 ~ 4.14)	0.013
Q3	16.68 (9.5 ~ 29.29)	<0.001	5.31 (2.99 ~ 9.45)	<0.001	3.95 (2.18 ~ 7.16)	<0.001
Q4	65.57 (37.77 ~ 113.82)	<0.001	13.50 (7.64 ~ 23.83)	<0.001	5.51 (3.03 ~ 10.03)	<0.001
*P-*trend	3.82 (3.47 ~ 4.22)	<0.001	2.39 (2.14 ~ 2.66)	<0.001	1.60 (1.42 ~ 1.81)	<0.001

Chinese cohort

Model 1: no covariates were adjusted.

Model 2: Gender, age, BMI were adjusted.

Model 3: Gender, age, BMI, ALP, GGT, ALT, AST, TB, ALB, DBIL, Cr, BUN, UA.

Japanese cohort

Model 1: no covariates were adjusted.

Model 2: Gender, age, BMI were adjusted.

Model 3: Gender, age, BMI, GGT, ALT, AST, TG, Exercise, Smoking status, Alcohol consumption, Diabetes.

BMI, Body mass index; ALB, albumin; ALP, Alkaline phosphatase; GGT, γ-glutamyl transpeptidase; AST, Aspartate aminotransferase; TG, Triglyceride; ALT, Alanine aminotransferase; LDL-C, Low-density lipid cholesterol; HDL-C, High-density lipoprotein cholesterol; TC, Total cholesterol; BUN, Serum urea nitrogen; UA, uric acid; TB, Total bilirubin; CHG, Cholesterol, high-density lipoprotein, and glucose; MASLD, Metabolic dysfunction-associated steatotic liver disease.

**Figure 1:**
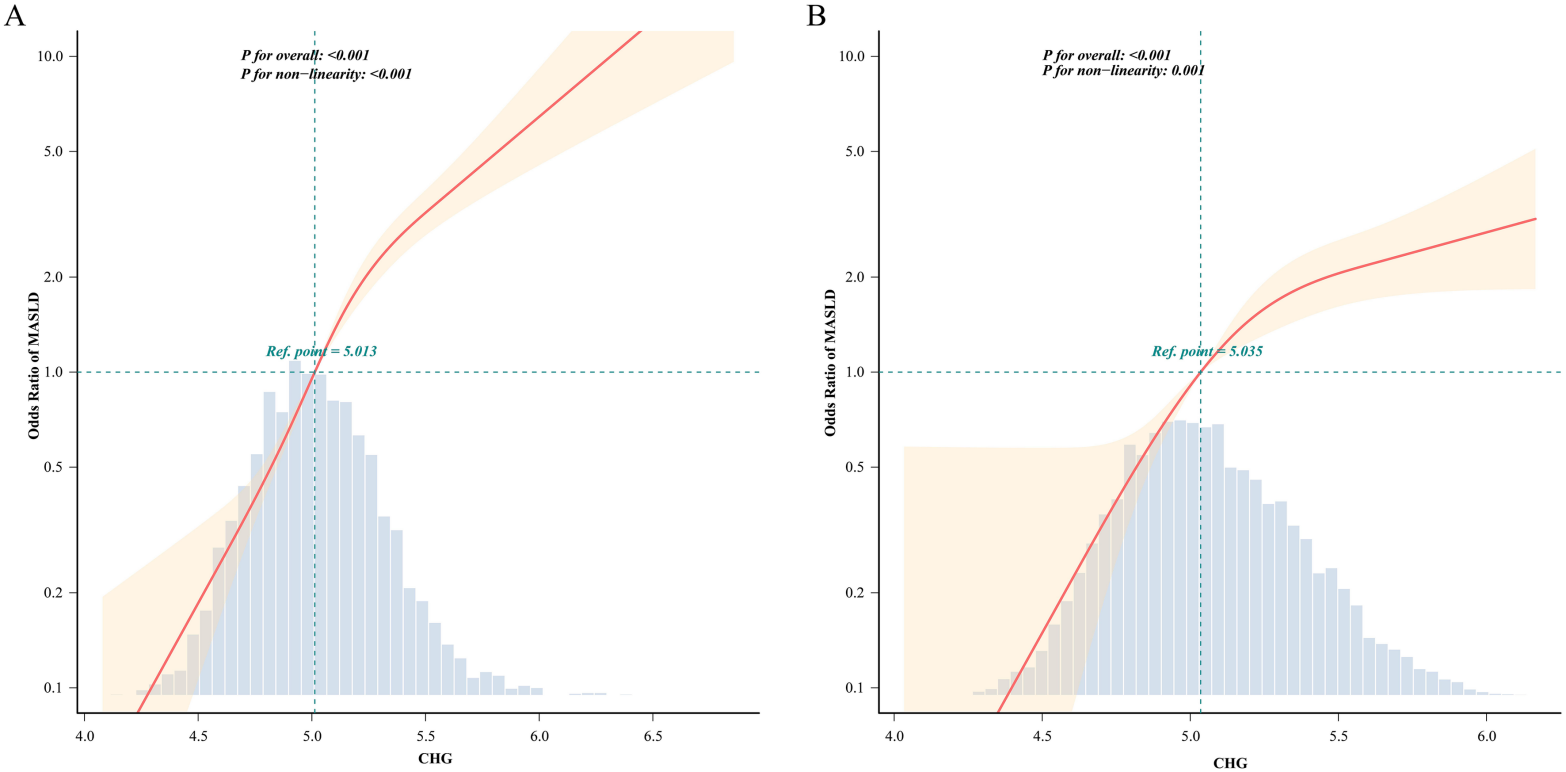
Dose–response relationship between CHG index and the odds of prevalent metabolic dysfunction-associated steatotic liver disease (MASLD) detected at baseline in two independent populations. Restricted cubic spline analyses with knots at the 5th, 35th, 65th, and 95th percentiles of CHG distribution showing the adjusted odds ratios (solid red line) and 95% confidence intervals (light orange shading) for MASLD according to CHG values. The reference line (dashed black line) represents an odds ratio of 1.0. **(A)** Chinese cohort (*n* = 16,173): fully adjusted for gender, age, BMI, ALP, GGT, ALT, AST, TB, TC, ALB, DBIL, Cr, BUN, UA. **(B)** Japanese cohort (*n* = 11,981): fully adjusted for gender, age, BMI, GGT, ALT, AST, TG, exercise, smoking status, alcohol consumption, diabetes.

Using the fully adjusted models, restricted cubic spline analyses were conducted to characterize the dose–response relationship between CHG and the odds of prevalent MASLD (Figure 1). In the Chinese cohort (Figure 1A), CHG was significantly associated with MASLD across the observed range (*P* for overall <0.001), with clear evidence of non-linearity (*P* for non-linearity <0.001). With CHG = 5.013 set as the reference (OR = 1.0), the odds of MASLD increased modestly at lower CHG values but rose more steeply once CHG exceeded approximately 5.0, with a progressively stronger increase at higher CHG levels. In the Japanese cohort (Figure 1B), a significant positive association was also observed (*P* for overall <0.001), again with evidence of non-linearity (*P* for non-linearity = 0.001). Using CHG = 5.035 as the reference point, the odds of MASLD increased sharply around the reference range and continued to rise thereafter, albeit with a more gradual slope at higher CHG levels. Overall, these spline curves support a monotonic increase in the odds of prevalent MASLD with increasing CHG in both populations.

### 3.3 ROC curve analysis for MASLD identification

**Figure 2:**
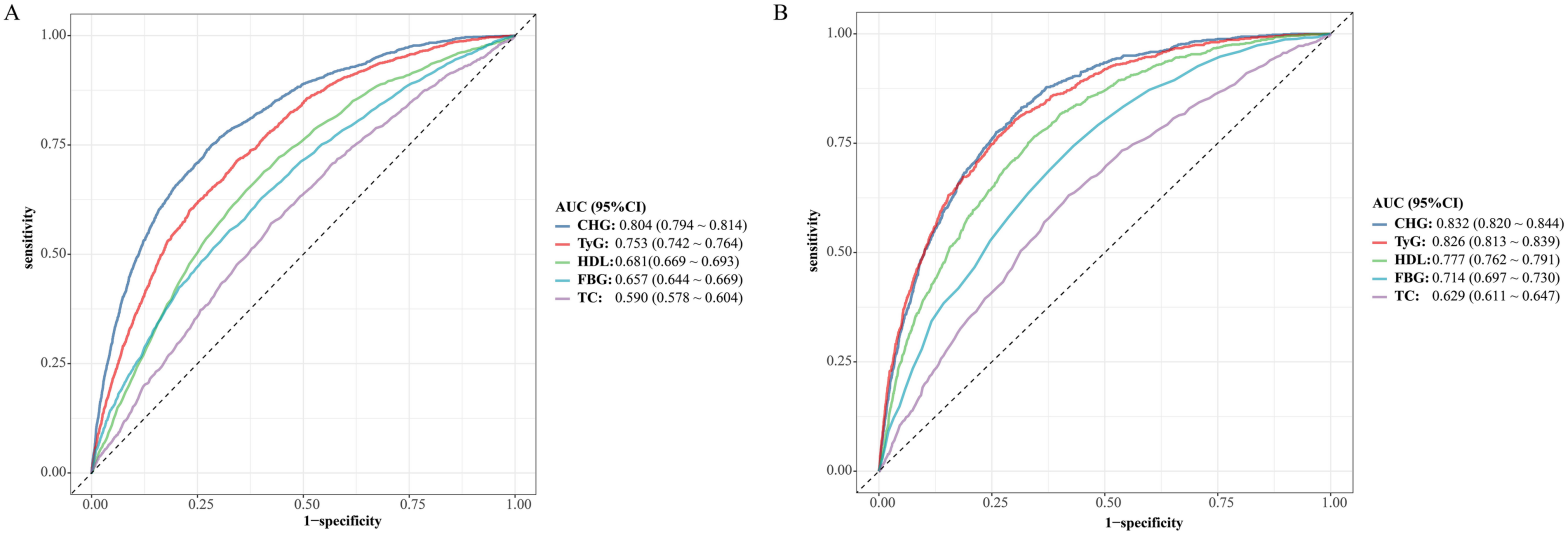
Receiver operating characteristic (ROC) curves comparing CHG with the triglyceride–glucose (TyG) index and traditional markers [HDL-C, fasting blood glucose (FBG), and total cholesterol (TC)] for identifying prevalent MASLD in two independent populations: **(A)** Chinese cohort (*n* = 16,173) and **(B)** Japanese cohort (*n* = 11,981). AUC (95% CI) values are provided in the legend; indicators are ordered by descending AUC, and a consistent color scheme is used across panels.

ROC analysis compared the discriminative ability of CHG with TyG, HDL-C, FBG, and TC for MASLD (Figure 2). In the Chinese cohort (Figure 2A), CHG achieved the highest AUC of 0.804 (95% CI 0.794–0.814), followed by TyG 0.753 (0.742–0.764), HDL-C 0.681 (0.669–0.693), FBG 0.657 (0.644–0.669), and TC 0.590 (0.578–0.604). In the Japanese cohort (Figure 2B), CHG also showed the highest AUC of 0.832 (0.820–0.844), marginally exceeding TyG 0.826 (0.813–0.839), with lower AUCs for HDL-C 0.777 (0.762–0.791), FBG 0.714 (0.697–0.730), and TC 0.629 (0.611–0.647). These findings suggest that CHG provides stronger discrimination for MASLD than single traditional lipid or glucose indices in both populations.

### 3.4 The contribution of CHG in MASLD discrimination

**Figure 3:**
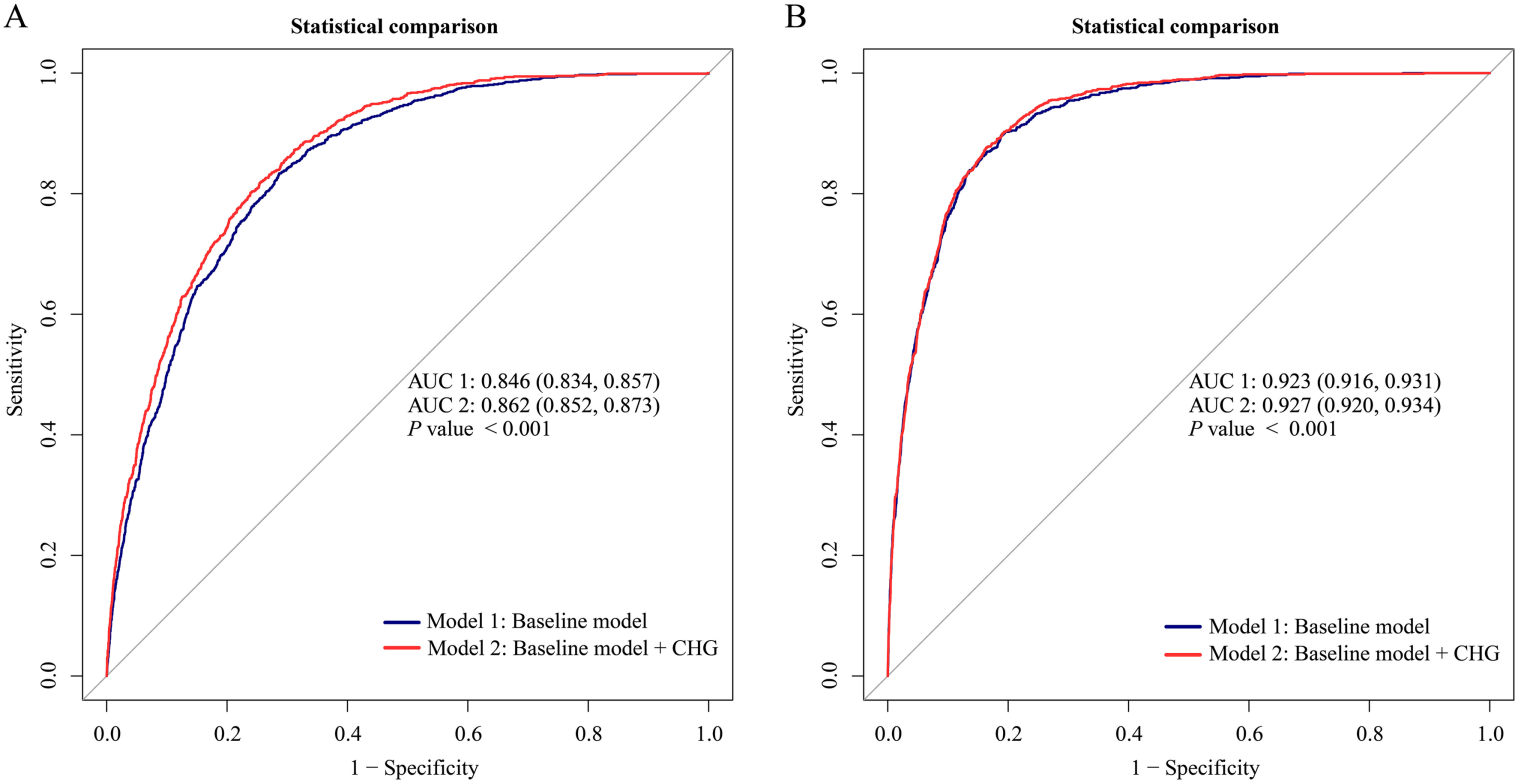
Incremental discriminative value of adding CHG to baseline models for identifying prevalent MASLD at baseline health check-ups. Receiver operating characteristic curves comparing the baseline model (blue line) and the baseline model plus CHG (red line). Predicted probabilities from cohort-specific multivariable logistic regression models were used to generate ROC curves. Baseline model covariates were as follows: Chinese cohort—sex, age, ALP, GGT, ALT, AST, ALB, TB, DBIL, BUN, Cr, UA, BMI; Japanese cohort—sex (gender), age, BMI, GGT, ALT, AST, TG, exercise, smoking status, alcohol consumption, diabetes. **(A)** Chinese cohort: AUC increased from 0.846 (95% CI 0.834–0.857) to 0.862 (95% CI 0.852–0.873) after adding CHG (*p* < 0.001). **(B)** Japanese cohort: AUC increased from 0.923 (95% CI 0.916–0.931) to 0.927 (95% CI 0.920–0.934) after adding CHG (*p* < 0.001).

To examine whether CHG improves discrimination beyond conventional risk factors, ROC curves were compared between the baseline model and the baseline + CHG model in both cohorts (Figure 3). In the Chinese cohort (Figure 3A), the AUC increased from 0.846 (95% CI 0.834–0.857) for the baseline model to 0.862 (95% CI 0.852–0.873) after inclusion of CHG (*p* < 0.001). In the Japanese cohort (Figure 3B), the AUC rose from 0.923 (95% CI 0.916–0.931) to 0.927 (95% CI 0.920–0.934) with CHG added to the baseline model (*p* < 0.001). Although the absolute gains in AUC were numerically small, the consistent improvement across both cohorts indicates that CHG provides additional discriminative information beyond traditional risk factors for MASLD.

### 3.5 Incremental discriminative value of CHG assessed by NRI and IDI

To evaluate the incremental value of CHG beyond the baseline model, NRI and IDI were calculated (Table 4). In the Chinese cohort, adding CHG to conventional risk factors resulted in an NRI of 0.5319 (95% CI: 0.4679–0.5959) and an IDI of 0.0368 (95% CI: 0.0299–0.0437). In the Japanese cohort, the corresponding estimates were 0.3421 for NRI (95% CI: 0.2761–0.4081) and 0.0063 for IDI (95% CI: 0.0025–0.0100). Collectively, these findings indicate that inclusion of CHG significantly improves MASLD risk reclassification and overall discriminative performance in both populations.

**Table 4:** Net reclassification improvement and integrated discrimination improvement analyses of CHG indices for MASLD identification.

Cohort	Baseline model + CHG vs. Baseline model			
	NRI (95%CI)	*P*-value	IDI (95%CI)	*P*-value
Chinese cohort	0.5319 (0.4679–0.5959)	<0.001	0.0368 (0.0299–0.0437)	<0.001
Japanese cohort	0.3421 (0.2761–0.4081)	<0.001	0.0063 (0.0025–0.0100)	0.0010

CHG, Cholesterol, high-density lipoprotein, and glucose; NRI, Net reclassification improvement; IDI, Integrated discrimination improvement; MASLD, Metabolic dysfunction-associated steatotic liver disease.

### 3.6 Subgroup analyses

**Figure 4:**
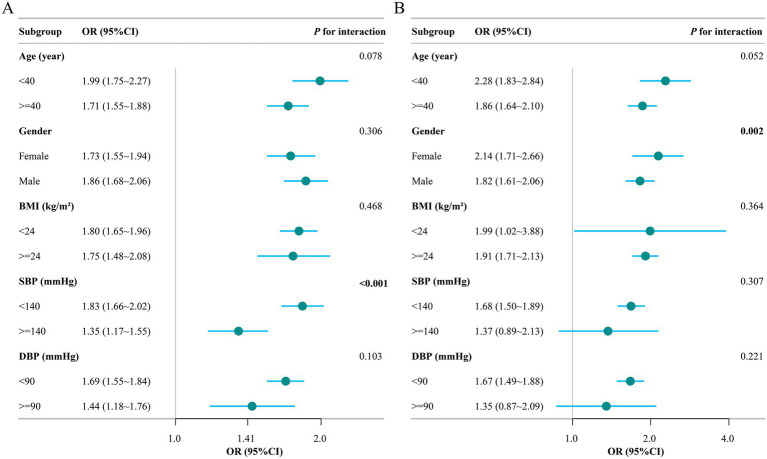
Subgroup analyses of the association between CHG and prevalent MASLD. Forest plots show adjusted odds ratios (ORs) and 95% confidence intervals for MASLD per 1-unit increase in CHG across strata of age, sex, BMI, systolic blood pressure (SBP), and diastolic blood pressure (DBP) in **(A)** the Chinese cohort and **(B)** the Japanese cohort. *p*-values for interaction were obtained from fully adjusted logistic regression models including a CHG × subgroup cross-product term.

Subgroup analyses stratified by age and sex (and additionally by BMI, SBP, and DBP) showed that the positive association between CHG and prevalent MASLD was directionally consistent across all strata in both cohorts (Figure 4). In the Chinese cohort, the association was similar across age, sex, BMI, and DBP strata (all *P* for interaction >0.05), whereas a significant interaction was observed for SBP (*P* for interaction <0.001), with a stronger association in participants with SBP < 140 mmHg. In the Japanese cohort, evidence of effect modification by sex was observed (*P* for interaction = 0.002), with a stronger association in females; interactions for BMI, SBP, and DBP were not statistically significant (all *P* for interaction >0.05), and age showed a borderline interaction (*P* for interaction = 0.052).

## 4 Discussion

This study evaluated the CHG index for identifying prevalent MASLD detected at baseline health check-ups in two independent Asian cohorts and yielded three main findings. First, higher CHG was strongly and dose-dependently associated with MASLD in both cohorts, with substantially higher adjusted odds in the highest quartile compared with the lowest. Second, CHG showed better discrimination than TC, FBG, HDL-C, and the TyG index, achieving the highest AUC in both populations. Third, adding CHG to baseline models provided incremental discriminative value, supported by improvements in reclassification metrics (NRI and IDI).

Although the AUC increase after adding CHG was statistically significant, its absolute magnitude was modest. This is expected when baseline models already have strong discrimination and because AUC can be relatively insensitive to incremental improvements. Therefore, we additionally reported NRI and IDI as complementary measures. The positive NRI and IDI indicate improved classification and greater separation of estimated probabilities between MASLD cases and non-cases, suggesting that CHG adds useful information beyond conventional predictors. Clinically, CHG may be most useful as a simple, low-cost adjunct for screening and baseline risk stratification in routine health check-ups, rather than as a stand-alone diagnostic test for MASLD.

A growing body of evidence suggests that routine lipid and glucose markers are closely related to MASLD. Studies in Chinese populations have reported associations of TC and HDL abnormalities with MASLD (11, 23), while FBG has also been identified as an independent factor linked to disease progression (24, 25). Notably, many earlier reports were conducted under historical NAFLD definitions, which are now encompassed within the contemporary MASLD framework; we cite these studies for context while using current MASLD terminology.

However, MASLD reflects coupled dysregulation of glucose and lipid metabolism, so traditional single markers such as TC, FBG, or HDL-C capture only isolated components and may underestimate overall metabolic risk. The CHG index, integrating TC, HDL-C, and FBG, has been proposed as a composite marker of metabolic dysfunction and insulin resistance (14). Prior studies in Chinese cohorts reported positive associations between CHG and fatty liver phenotypes (26), but evidence specific to MASLD and across ethnic groups has remained limited. Our study addresses this gap by demonstrating that CHG is independently associated with MASLD and consistently outperforms each single component in both Chinese and Japanese cohorts. In support of its broader metabolic relevance, an independent SHAP-based analysis from another study identified CHG as a key contributor to ASCVD risk stratification among MASLD patients (27), suggesting that CHG captures clinically meaningful systemic risk.

Based on current research, several pathophysiological mechanisms may explain the relationship between blood glucose and lipid levels and MASLD. MASLD is closely associated with metabolic dysfunction, with insulin resistance (IR) playing a central role in its pathogenesis (28). IR is a primary driver of dyslipidaemia in fatty liver disease (29), and dyslipidaemia in MASLD has been shown to correlate with markers of impaired insulin sensitivity (30). As a key hormone regulating lipid metabolism, insulin influences triglyceride homeostasis and HDL-C levels through multiple mechanisms (31). Because CHG incorporates HDL-C, a pivotal indicator of lipid metabolism, it is biologically relevant to hepatic fat accumulation (32, 33). Moreover, high-fat intake increases the risk of dyslipidaemia (34) and promotes hepatic lipid accumulation, leading to elevated free fatty acids, free cholesterol, and other lipotoxic metabolites that trigger hepatocellular injury and inflammatory responses, thereby facilitating MASLD progression (35, 36). In addition, compensatory hyperinsulinemia can further enhance hepatic triglyceride synthesis, promoting steatosis, steatohepatitic inflammation, and progressive liver injury (37). Although the pathogenic pathways of MASLD are heterogeneous, this IR driven glucose-lipid dysregulation framework provides a plausible basis for the observed associations.

The findings have clear clinical implications. CHG is inexpensive and readily available from routine blood tests, so it can be integrated into standard health examinations and used for large-scale MASLD screening. As a composite index combining lipid and glycaemic information, CHG offers a more holistic reflection of the metabolic dysfunction underlying MASLD than any single marker alone. Given its superior discriminative performance in both cohorts, CHG may serve as a practical first-line tool to identify individuals more likely to have MASLD at baseline for further evaluation and earlier intervention in routine clinical practice.

A major strength of this work is the use of two separate cohorts analyzed in parallel, which allowed us to test the reproducibility of CHG across different settings and populations. The large sample sizes in both cohorts provided stable estimates and enabled clear evaluation of dose response patterns. We assessed CHG using several layers of evidence, including multivariable regression, non-linearity checks, discrimination metrics, and reclassification analyses, giving a well-rounded view of its discriminative utility. The head-to-head comparison with routine single markers also clarifies the practical gain of using an integrated index in MASLD risk assessment.

Several limitations warrant attention. The cohorts were drawn from Asian health checkup populations, so applicability to other ethnicities and clinical settings requires confirmation. Ethnic differences in lipid metabolism, insulin resistance patterns, and body fat distribution may influence both the distribution of CHG and MASLD phenotype, including the relatively common lean/non-overweight MASLD phenotype in Asian populations. Therefore, CHG operating characteristics and optimal cut-offs derived from Asian cohorts should not be assumed to transfer directly to non-Asian populations, and external validation with potential recalibration, such as population-specific thresholds or model recalibration, may be required before broader clinical implementation.

To align with contemporary diagnostic principles, MASLD was operationally defined using available health check-up variables (ultrasonographic steatosis together with at least one cardiometabolic risk condition); however, residual misclassification remains possible. MASLD was diagnosed by ultrasound rather than biopsy, which may introduce misclassification and underestimate prevalence, and differences in imaging practice between cohorts could affect comparability (38, 39). The retrospective, observational design based on secondary datasets is subject to information bias and residual confounding from unmeasured factors such as diet, detailed activity patterns, genetics, and environmental exposures. Because this is a retrospective cross-sectional analysis based on baseline ultrasonography, our findings reflect discrimination for prevalent MASLD detected at baseline rather than prediction of future incident disease; prospective cohort studies are warranted to evaluate the predictive value of CHG for incident MASLD. Our findings are derived from BMI ≤ 25 kg/m^2^ cohorts and may not directly generalize to overweight/obese populations; further validation across the full BMI spectrum is warranted. Finally, these analyses establish association and discrimination performance but cannot prove causality or determine whether lowering CHG would improve MASLD outcomes, highlighting the need for prospective and causal studies.

## 5 Conclusion

Higher CHG was consistently and dose-dependently associated with MASLD in both Chinese and Japanese cohorts. CHG outperformed TC, FBG, HDL-C, and TyG, and added discriminative value to baseline models for identifying MASLD, supporting its use as a simple, low-cost marker for MASLD screening and baseline risk stratification. Further prospective and causal studies are needed to confirm generalizability and clinical impact.

## Data Availability

The original contributions presented in the study are included in the article/Supplementary material, further inquiries can be directed to the corresponding authors.

## Footnotes

1
https://datadryad.org/
